# UV-Heat Treatments for the Control of Foodborne Microbial Pathogens in Chicken Broth

**DOI:** 10.1155/2015/436030

**Published:** 2015-10-11

**Authors:** M. Gouma, E. Gayán, J. Raso, S. Condón, I. Álvarez

**Affiliations:** ^1^Tecnología de los Alimentos, Facultad de Veterinaria, Universidad de Zaragoza, 50013 Zaragoza, Spain; ^2^Department of Microbial and Molecular Systems (M^2^S), Laboratory of Food Microbiology, KU Leuven, 3001 Leuven, Belgium

## Abstract

This investigation established the process criteria for using UV-C light and mild heat (UV-H treatment) to inactivate 5-Log_10_ cycles (performance criterion) of common foodborne pathogen populations, *Escherichia coli*, *Salmonella* Typhimurium, *Listeria monocytogenes*, and *Staphylococcus aureus*, when inoculated in chicken broth. To define the target microorganism and the proper UV-H treatment conditions (including UV dose, treatment time, and temperature) that would achieve the stated performance criterion, mathematical equations based on Geeraerd's model were developed for each microorganism. For the sake of comparison, inactivation equations for heat treatments were also performed on the same chicken broth and for the same microorganisms. *L. monocytogenes* was the most UV-H resistant microorganism at all temperatures, requiring a UV dose between 6.10 J/mL (5.6 min) and 2.26 J/mL (2.09 min) to achieve 5-Log_10_ reductions. In comparison with UV treatments at room temperatures, the combination of UV and mild heat allowed both the UV dose and treatment time to be reduced by 30% and 63% at 55°C and 60°C, respectively. Compared to heat treatments, the UV-H process reduced the heating time for 5-Log_10_ reductions of all the investigated microorganisms in chicken broth from 20-fold to 2-fold when the operating temperature varied from 53 to 60°C.

## 1. Introduction

Despite the fact that conventional heat treatments still prevail in the food industry, several nonthermal food processing technologies have emerged recently. Compelled by the strong preference of consumers for fresh and minimally processed food, research into food processing has focused on the investigation of alternatives to pasteurization, which has long been popular for its ability to ensure microbial inactivation at lower temperatures while minimizing losses of the organoleptic and nutritional properties of food. One of these technologies is short wave ultraviolet radiation (UV-C), which has numerous advantages, including the ability to inactivate a wide range of pathogenic and spoilage microorganisms in juices [1, 2], thereby minimizing the loss of nutritional and sensorial quality [3]. Moreover, UV-C does not generate chemical residues or toxic compounds [4] and requires very little energy consumption compared with other nonthermal pasteurization processes [5]. The germicidal properties of UV-C light rely on DNA's absorption of the UV light, which induces structural distortions in the DNA molecule, inhibiting transcription and replication and eventually leading to cell death.

Although previous studies have shown that UV-C radiation can be an effective method to inactivate microorganisms in liquid food, practical applications of this technology are limited due to its low penetration capacity in liquids with high absorption coefficient and turbidity. It is well known that the optical properties of the treatment medium strongly influence the lethal effect of UV light [6] due to absorption, reflection, scattering, and refraction phenomena caused by the presence of color compounds and soluble or suspended particles. In order to overcome these limitations, combinations of UV radiation with other nonthermal technologies, such as pulsed electric fields, have been designed, based on hurdles technology approach. For instance, it has been demonstrated that UV light followed by pulsed electric fields (PEF) treatments has an additive effect [7].

Recently, we have demonstrated that the simultaneous application of UV radiation and heat at sublethal temperatures (UV-H treatment) remarkably enhances the former's microbial inactivation capacity. Specifically, studies on the inactivation of various bacterial species, both in buffers and fruit juices (orange and apple), have shown that UV-H treatments result in a synergistic lethal effect, with the temperature of maximum synergism being different for each bacterial species [8–10]. However, so far, our studies have focused on low pH media, where acidity can reduce the heat resistance of bacteria and, consequently, enhance the effectiveness of UV-H treatments. Additionally, data on food with more neutral pH are scarce but do include some studies on egg white [11–13]. Therefore, the question of whether a similar synergistic lethal effect could also occur in other liquid foods with different pH, chemical composition, and absorption coefficients was raised.

To challenge the inactivation effect of UV-H treatments on complex food matrices, chicken broth was chosen as a multi-ingredient liquid food with higher pH and a more complex chemical composition that provides a favorable environment for bacteria. The goal of this study was to investigate whether the UV-H combined process can be applied to liquid food that has a high absorption coefficient, turbidity, and low acidity such as chicken broth, resulting in a synergistic lethal effect comparable with/similar to that which has been obtained in the case of fruit juices. For this study, we have used the most UV resistant strains of different pathogenic bacteria as reference microorganisms, such as* Escherichia coli*,* Salmonella* Typhimurium,* Listeria monocytogenes*, and* Staphylococcus aureus*.

## 2. Materials and Methods

### 2.1. Bacterial Culture and Media

The strains of* E. coli* STCC 4201,* Salmonella* Typhimurium STCC 878,* L. monocytogenes* STCC 5672, and* S. aureus* STCC 4465 were obtained from the Spanish Type Culture Collection (STCC). The bacterial cultures were maintained frozen at –80°C in cryovials. A broth subculture was prepared by inoculating 10 mL of tryptone soy broth (Biolife, Milan, Italy) supplemented with 0.6% (w/v) yeast extract (Biolife) (TSBYE) with a loopful of growth from tryptone soy agar (Biolife) supplemented with 0.6% (w/v) yeast extract (TSAYE). The subculture was incubated at 35°C for 6–12 h in a shaking incubator (150 rpm; Heidolph Instruments, Vibramax 100, Schwabach, Germany). With these subcultures, 250 mL flasks containing 50 mL of TSBYE were inoculated to reach a concentration of 10^4^ CFU/mL and then incubated for 24 h under the same conditions until the stationary growth phase was reached (2 × 10^9^ CFU/mL).

### 2.2. UV Equipment and Treatments

UV treatments were carried out in a unit with 8 individual annular thin film flow-through reactors connected in a series and equipped with a feed tank and a peristaltic pump (ISM 10785, Ismatec, Glattbrugg, Switzerland), as described previously by Gayán et al. [8]. Each reactor included a low-pressure mercury vapor lamp (8 W of input power; model TUV 8WT5, Philips, USA), which converted 30% of the input power as UV-C radiation (Philips Electronics, 2012), emitting 85% of UV-C energy at 254 nm. The lamp was attached to the axis of an outer glass tube (25 mm of inner diameter) and was enclosed using a quartz tube (20 mm of outer diameter) to prevent direct contact of the lamp with the treatment medium. In the annular gap (2.5 mm), a stainless steel coil spring was installed to improve the flow's turbulence. The outside and inside coil diameters of the spring were 23 mm and 25 mm, respectively, and its length and pitch were 270 mm and 10 mm, respectively. A manual sampling valve was located in the outlet of each reactor. The entire unit was submerged in a 90 L water bath (25.0–60.0°C) heated by the circulating water of a peripheral thermostatic bath (Kattebad K12, Huber, Offenburg, Germany). The equipment also included a heating/cooling coil exchanger at the inlet of the first reactor. Thermocouples (ZA 020-FS, Almeco, Bernburg, Germany) fitted to the inlet and outlet of the first and last reactor, respectively, allowed for treatment temperature control.

Chicken broth (absorption coefficient = 19.6 cm^−1^, turbidity = 4460 NTU, pH = 5.2) for use as treatment medium was purchased from a local market (Interal S.A., Spain). Broth's absorption coefficient was measured spectrophotometrically (254 nm; UV500, Unicam Limited, Cambridge, UK). Samples were diluted and evaluated using quartz cuvettes (Hellma, Müllheim, Germany) with path lengths of 1 mm, 2 mm, and 10 mm. The absorption coefficient of the diluted samples was determined from the slope of the absorbance versus the path length and corrected by the dilution factor. Turbidity was measured with a nephelometer (HI 83749, Hanna Instrument, Szeged, Hungary). pH was measured using a pH-meter Basic 20 (Crison Instrument, Barcelona, Spain). The chicken broth was inoculated with the bacterial suspension to achieve 10^5^–10^7^ CFU/mL and pumped (8.5 L/h) through the heat exchanger to the reactors. When the treatment conditions were stabilized, samples were withdrawn through the sampling valves and 0.1 mL or 1 mL was immediately pour-plated into the recovery medium.

### 2.3. Heat Treatments

Heat treatments were carried out in specially designed thermoresistometer TR-SC [14]. This instrument consisted of a 400 mL vessel with an electrical heater for thermostation, an agitation device used to ensure inoculum distribution and temperature homogeneity, a pressurization system, and ports for injecting the microbial suspension and for extraction of samples. Once the preset temperature had attained stability (*T* ± 0.05°C), 0.2 mL of an adequately diluted microbial cell suspension was inoculated into the vessel, which contained the 350 mL of chicken broth. After inoculation, 0.2 mL samples were collected at different heating times and were immediately pour-plated.

### 2.4. Incubation of Treated Samples and Survival Counting

TSAYE was used as a recovery medium and the plates were incubated at 35°C for 24 h for* E. coli*,* Salmonella* Typhimurium, and* S. aureus* and for 48 h for* L. monocytogenes*. After incubation, colony forming units (CFU) were counted using an improved Image Analyzer Automatic Colony Counter (Protos, Synoptics, Cambridge, UK), as described elsewhere [15].

### 2.5. Curve Fitting and Dose Calculation

Survival curves were obtained by plotting the logarithm of the survival fraction* versus* UV dose (*d*) expressed in joules per milliliter and time (*t*) expressed in minutes for UV and heat treatments, respectively. To compare UV-H treatments with thermal treatments, UV-H survival curves were also expressed in treatment time. The UV dose delivered to the treatment medium was estimated with a chemical dosimeter. To this end, the iodide-iodate actinometer (quantum yield = 0.73 ± 0.02) was used, following the indications of Rahn et al. [16]. The actinometer buffer was pumped through the installation at 8.5 L/h and the increase in absorbance (352 nm) was determined at the outlet of each reactor [8]. From this data, the photon flux (254 nm), as received per second by each volume fraction of the treatment medium, was estimated according to Montalti et al. [17]. Thus, considering the energy of a photon at 254 nm, the UV dose delivered to each reactor was 0.49 J/mL.

To fit the survival curves obtained at each temperature and to calculate resistance parameters, the GInaFiT inactivation model-fitting tool was used [18]. Specifically, the log-linear regression plus shoulder model from Geeraerd et al. [19] was chosen (1) since most of the survival curves exhibited shoulders. This model describes the survival curves through two parameters: the shoulder length (Sl), defined as dose or time before the exponential inactivation begins, and the inactivation rate (*K*_max⁡_), defined as the slope of the exponential part of the survival curve. *N*_0_ and *N*_*t*_ represent the initial numbers of the microbial population and the number of microorganisms that survive at the end of the treatment time (*t*), respectively. Consider the following equation:

(1) $$\begin{array}{c} N_{t}=N_{0}e^{-K_{\max}\text{Sl}}\left(\frac{e^{K_{\max}\text{Sl}}}{1+\left(e^{K_{\max}\text{Sl}}-1\right)e^{K_{\max t}}}\right). \end{array}$$

To describe the relationship between treatment temperature (*T*) and Sl and *K*_max⁡_ parameters, mathematical equations based on the Weibull distribution were chosen. For Sl, the equation introduced by Albert and Mafart [20] (2) was used as a secondary model, whereas the thermodependence of *K*_max⁡_ was described using the mirror image of the Mafart [21] model (3):

(2) $$\begin{array}{c} {\text{Sl}}_{T}=\left({\text{Sl}}_{0}-{\text{Sl}}_{\text{res}}\right)10^{-{\left(t/\delta \right)}^{p}}+{\text{Sl}}_{\text{res}}, \end{array}$$

(3) $$\begin{array}{c} K_{\max T}=\left\lfloor K_{\max0}10^{-{\left(t/\delta \right)}^{p}}\right\rfloor , \end{array}$$

where Sl_*T*_ and *K*_max⁡*T*_ are the shoulder length and the inactivation rate of UV-H treatments at temperature *T*, respectively; Sl_0_ and *K*_max⁡0_ are the shoulder length and the inactivation rate of the survival curves of UV treatments at room temperature, respectively; and Sl_res_ is the residual shoulder when the treatment temperature was increased. *δ* and *p* are, respectively, the scale and shape parameters. The *δ* value represents the temperature increase necessary to achieve the first decimal reduction of Sl or *K*_max⁡_ (from Sl_0_ and *K*_max⁡0_ to Sl_0_/10 and *K*_max⁡0_/10). The *p* parameter (*p* > 1) accounts for the profile of the downward concavity of curves [20, 21]

For the heat survival curves, which showed an initial shoulder phase, the Geeraerd model was also used as a primary model. In order to study the relationship between the inactivation model parameters and the treatment temperature, Albert and Mafart's equation [20] was used for Sl, as described above, and simple log-linear equations were used for *K*_max⁡_, while the slope and the intercept of the regression line were considered model parameters. The coefficient of determination (*R* ^2^), the root mean square error (RMSE), the bias (*B*_*f*_), and accuracy (*A*_*f*_) factors were used to determine the goodness of fits of both the primary and the secondary models, as well as the accuracy of the final equations [22]. The bias factor indicates systematic over-(*B*_*f*_ > 1) or under-(*B*_*f*_ < 1) prediction of the observed data. On the other hand, the accuracy factor indicates the extent to which the predictions differ from the observed data.

### 2.6. Statistical Analyses

Statistical analyses, a *t*-test, and an ANOVA test were carried out using the GraphPad PRISM 5.0 software (GraphPad Inc., San Diego, CA, USA) and differences were considered significant for *P* ≤ 0.05. All microbial resistance determinations, as well as analytical assays, were performed at least three times on different workings days. The error bars in the figures correspond to the mean standard deviation.

## 3. Results and Discussion

This study investigated the thermodependence of the UV inactivation of pathogenic bacteria in chicken broth. The intention was to establish the UV-H treatment conditions (process criteria) necessary for obtaining a certain level of inactivation of the pathogenic microorganisms of reference. For this purpose, the effect of temperature on the UV lethality of UV tolerant strains of* E. coli* (STCC 4201),* Salmonella* Typhimurium (STCC 878),* L. monocytogenes* (STCC 5672), and* S. aureus* (STCC 4465) was assessed. The strains used in this investigation were the most UV resistant among five different strains of each investigated microorganism according to previous studies [8, 23–25] carried out under the same methodology. In particular, the selected strains of* E. coli*,* Salmonella* Typhimurium,* L. monocytogenes*, and* S. aureus* were 15.6, 15.1, 15.8, and 2.7% more resistant than the second most resistant strain of each microorganism.

To describe this influence and to define the process criteria, mathematical equations, including UV dose, time, and temperature, have been developed. In order to compare results, the heat resistance of the indicated microorganisms was also investigated and modeled.

### 3.1. Microbial Inactivation by UV-H Treatments in Chicken Broth

**Figure 1:**
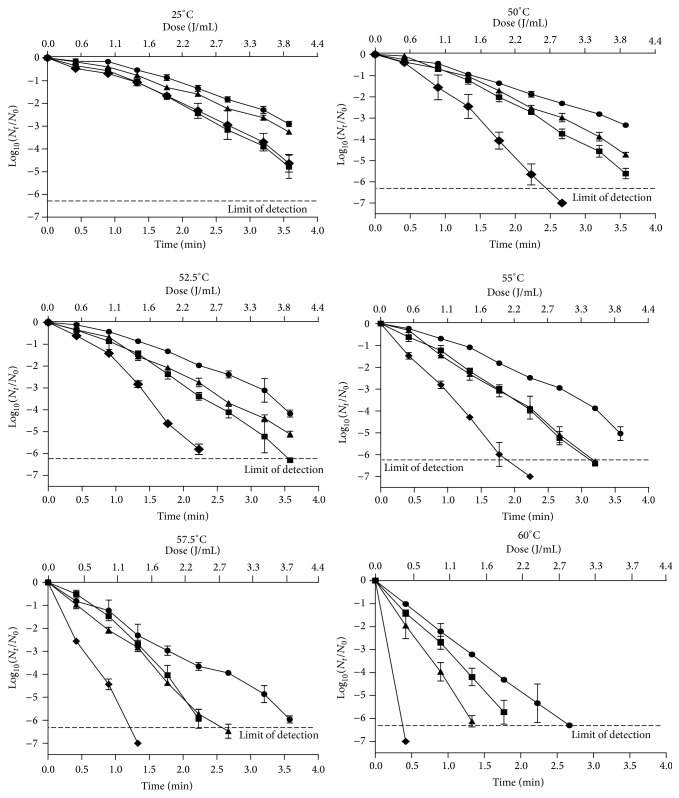
Survival curves of* E. coli* (STCC 4201) (■),* Salmonella* Typhimurium (STCC 878) (▲),* L. monocytogenes* (STCC 5672) (●), and* S. aureus* (STCC 4465) (◆) to UV treatment at room temperature (25°C) and to combined UV-H treatments at 50.0°C, 52.5°C, 55.0°C, 57.5°C, and 60.0°C in chicken broth.

The survival curves of* E. coli*,* Salmonella* Typhimurium,* L. monocytogenes*, and* S. aureus* to UV treatments at room temperature and at 50.0, 52.5, 55.0, 57.5, and 60.0°C (UV-H) are presented in Figure 1. An initial lag phase (shoulder) was observed for most of the survival curves. After this, the microbial death followed a logarithmic order, but no tailing took place. UV-H inactivation curves displayed shoulder phases, which are often observed in survival curves for UV-C light [26, 27]. According to the “multihit target theory,” shoulders are related to the DNA damage and repair phenomena [28]. DNA repair systems can repair damage up to certain UV doses, resulting in shoulders. Once the maximum DNA repair capability is surpassed, additional UV exposure is lethal for microorganisms and survivors exponentially decline [29].

When the maximum UV dose possible in one pass through the equipment (3.92 J/mL) was applied, UV treatments at room temperature (25°C) decreased the microbial population of the investigated bacteria from 5-Log_10_ cycles for* E. coli* and* S. aureus* to 3-Log_10_ cycles for* Salmonella* Typhimurium and* L. monocytogenes*, which showed the highest UV resistance (Figure 1). This level of inactivation for the same bacteria was higher than that observed in other products like fruit juices that have lower turbidity but higher absorption coefficient. Thus, Gouma et al. [30] observed hardly 1-Log_10_ reductions of these microorganisms in apple juice (turbidity 7.4 NTU, *α* = 24 cm^−1^) after applying the same UV treatment at 25°C in the same facility. Since pH did not affect the UV lethality [23] and since the turbidity of the chicken broth (4460 NTU) was higher than that of the apple juice, the larger inactivation observed in this investigation could be due to the slightly lower absorption coefficient of the chicken broth (19.6 cm^−1^) compared to that of the apple juice. This lower absorption coefficient would cause less UV-C light to be absorbed by the treatment medium, meaning that the same UV dose would have more bactericidal effect in the chicken broth than in the apple juice [8]. Also, any component of the chicken broth could interact on the microbial UV resistance, thereby increasing the UV lethality.

Although 3-Log_10_ cycles of inactivation were achieved after the maximum applied UV dose at 25°C for all the investigated pathogenic microorganisms, which were selected in previous studies due to their being the most UV resistant [8, 23–25], a reduction of 99.9% of the microbial population could not be sufficient to ensure the safety of chicken broth; for example, at least 5-Log_10_ reductions of the pathogen of reference are necessary for the pasteurization of fruit juices [31]. Therefore, it would be necessary to increase the lethality of UV treatments.

The application of UV at moderate temperatures has resulted in a synergistic increase of UV lethality [11, 13]. In this investigation, when the treatment temperature was raised between 50.0°C and 60.0°C (Figure 1), the UV inactivation of all investigated microorganisms improved considerably. For instance, the UV inactivation of the most resistant microorganism,* L. monocytogenes*, with a dose of 2.45 J/mL (2.23 min), increased from 1.34-Log_10_ cycles at 25°C to 1.87, 1.96, 2.48, 3.66, and 5.35-Log_10_ cycles at 50.0°C, 52.5°C, 55.0°C, 57.5°C, and 60.0°C, respectively. These results indicated that combining UV light with mild heat increased the UV inactivation of microorganisms in chicken broth, which paralleled the observation of a higher effect of UV and heat in other food products. This fact suggests the possibility of designing a feasible UV-H hygienization process for this kind of product (i.e., broth), which is, apparently, difficult to treat with this technology due to its high turbidity and absorption coefficient.

Although an increment of the UV lethality has been observed when augmenting the temperature, the different behavior of* E. coli*,* Salmonella* Typhimurium,* L. monocytogenes*, and* S. aureus* in response to UV-H treatments in relation to the treatment temperature makes it difficult to compare data. Moreover, the obtained survival curves showed shoulders at lower temperatures that disappeared when the temperature was increased, which limited the application of simpler first order inactivation kinetics. Therefore, it was necessary to develop mathematical models that enabled an evaluation of the effect of temperature on UV lethality in chicken broth for each investigated microorganism.

To describe UV-H inactivation kinetics, the log-linear regression plus the shoulder model of Geeraerd et al. [19] (1) (primary model) was used because it allowed the length of the shoulders and the log-linear rate of inactivation to be accurately and independently described.  Table 1 includes the averages and the standard deviations of the model parameters (*K*_max⁡_ and Sl), expressed in time terms, obtained from the fitting of the UV-H survival curves of all microorganisms tested at different temperatures. The coefficient of determination (*R* ^2^) and the root mean square error (RMSE) values are also included to illustrate the goodness of the fits. As observed, the UV lethality improved when the treatment temperature was raised and stemmed from the decrease of the shoulder phase (Sl) of the UV-H survival curves until it disappeared. In addition, the slope of the survival curves (*K*_max⁡_) increased with the rising temperature. Furthermore, the UV resistance variability between species was maintained at different temperatures and was reflected in the Sl and *K*_max⁡_ values. Thus, the higher resistance of* L. monocytogenes* was due to larger values of Sl and smaller values of *K*_max⁡_. On the contrary,* S. aureus* was the most sensitive microorganism, showing smaller Sl values and higher *K*_max⁡_ ones. Finally, the Gram negative bacteria* E. coli* and* Salmonella* Typhimurium showed a similar behavior and Sl and *K*_max⁡_ values. In general, it is believed that Gram positive bacteria are more UV resistant than Gram negative, which can be attributed to the thicker peptidoglycan cell wall of the former [32, 33]. In this study, the UV resistance of* S. aureus* at 25°C was similar to that of* E. coli* and slightly lower than that of* Salmonella* Typhimurium, which demonstrates that this statement should not be considered a general rule. In fact, other authors have reported a higher susceptibility of* S. aureus* to UV technologies compared with coliforms [34, 35]. At higher temperatures, that statement cannot be maintained since heat interferes on the microbial UV resistance. For example, the highest UV-H lethality for* S. aureus* would be due to the higher heat sensitivity of this microorganism compared with the other investigated bacteria as it will presented later on.

**Figure 2:**
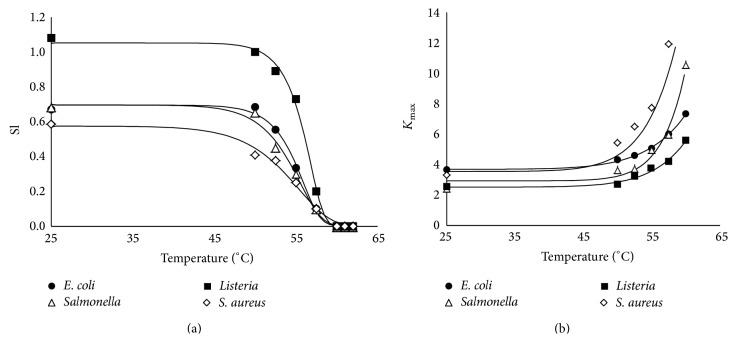
Relationships between temperature and Sl (a) or *K*_max⁡_ (b) values obtained after the fitting of Geeraerd's model (1) to the UV-H inactivation data of* E. coli* (●),* Salmonella* Typhimurium (∆),* L. monocytogenes* (■), and* S. aureus* (⋄) in chicken broth. Solid lines represent the fitting curves for Sl and *K*_max⁡_ calculated from Albert and Mafart's (2) and Mafart's equation (3), respectively.

To describe the effect of the changes in temperature on the kinetic parameters obtained after fitting the primary model to the UV-H inactivation data of* E. coli*,* Salmonella* Typhimurium,* S. aureus*, and* L. monocytogenes* in chicken broth (shown in Table 1), the corresponding secondary models for each microorganism were developed.  Figure 2 depicts the relationship between temperature and the Sl and *K*_max⁡_ values for all the investigated microorganisms. Mathematical equations (secondary models) based on the Weibull distribution—Albert and Mafart's equation (2) for Sl and Mafart's equation (3) for *K*_max⁡_—were used to describe the thermodependence of both parameters.  Table 2 compiles the obtained parameters (*δ*, *p*, Sl_0_, Sl_res_, and *K*_max⁡0_) from the secondary models of Sl and *K*_max⁡_ for each microorganism, including the *R* ^2^ and RMSE values from the fits. The relationship between the Sl (Figure 2(a)) and the temperature displayed a sigmoid profile for all microorganisms, first showing a lag phase and then dropping off to zero.* L. monocytogenes* showed higher shoulder length values of the UV-H survival curves than all the other species at all treatment temperatures tested, especially at temperatures ranging from 25°C to 55.0°C. Above this value, differences were reduced until the shoulder length became zero. When *K*_max⁡_ values were plotted against treatment temperature, concave upward curves were observed (Figure 2(b)). The inactivation rate of* S. aureus* and* Salmonella* Typhimurium was more sensitive to temperature changes than that of* E. coli* and* L. monocytogenes*. This behavior was evidenced in the scale parameter (*δ*) of *K*_max⁡_ secondary models, which determined the temperature increment that reduced the *K*_max⁡_ 10-fold (Table 2). *δ* values for* S. aureus* and* Salmonella* Typhimurium were smaller than those obtained for* L. monocytogenes* and* E. coli*.

**Figure 3:**
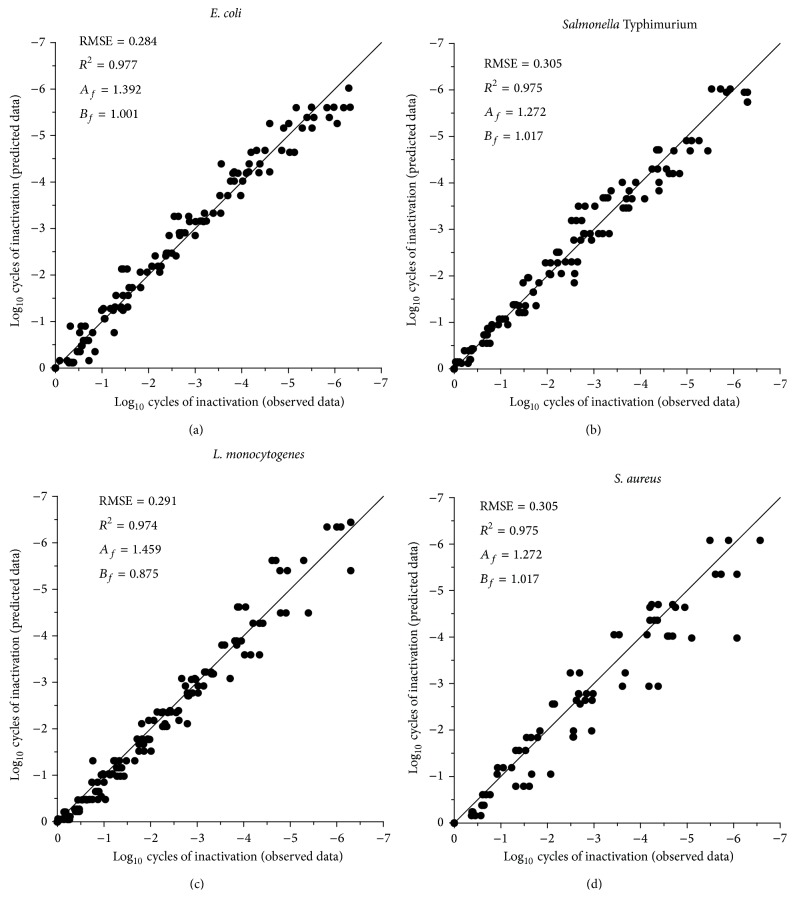
Correlation between observed and predicted data obtained from the tertiary models for* E. coli* (a),* Salmonella* Typhimurium (b),* L. monocytogenes* (c), and* S. aureus* (d) when treated by UV-H process. *R*_2_, RMSE, accuracy (*A*_*f*_), and bias (*B*_*f*_) factors from each prediction are also indicated in the figures.

To describe, compare, and predict the microbial inactivation by UV-H treatments of* E. coli*,* Salmonella* Typhimurium,* L. monocytogenes*, and* S. aureus* in chicken broth, final equations were developed. These equations were obtained by introducing the secondary models for Sl and *K*_max⁡_ values (2) and (3) into Geeraerd's primary model (1). To show the goodness of the fits of the final equations, Figure 3 presents the plots of the observed versus predicted data by the final equations for each microorganism. The difference between a point on the graph and the line of equivalence is a measure of the accuracy of the corresponding estimation. The *R* ^2^, RMSE, accuracy (*A*_*f*_), and bias (*B*_*f*_) factors from each prediction were also indicated in the figures. The calculated values demonstrated that, in general, the final equations accurately predicted the UV-H inactivation of all microorganisms without observing over or under predictions. Therefore, the developed final equations would be adequate to compare the UV-H resistance of the different microorganisms and to define the processing conditions (process criteria) to achieve a certain level of pathogenic microbial inactivation (performance criteria), as will be discussed later on.

It has been demonstrated that the improvement of UV-H inactivation was due to the occurrence of a synergistic lethal effect and that the magnitude of this effect increased when the treatment temperature was raised to a threshold value [8, 10]. Above this temperature, thermal lethal effects began to predominate over UV lethality, and UV-H synergism was reduced to the point of disappearing, whereby microbial death was then exclusively due to heat. Prior studies on the combined UV-H treatment of apple juice have shown that* E. coli* STCC 4201 displayed the maximum UV-H synergism at a treatment temperature of about 55°C; above this temperature, the synergism decreased until it disappeared at 60.0°C when UV-H and heat survival curves overlapped [10]. Therefore, to take advantage of the combined UV-H process, the treatment temperature should be limited to temperatures below the intersection of UV-H and heat lethality. This requires knowledge of the heat resistance abilities of target microorganisms and their thermodependence. Therefore, the next step was to investigate the heat resistance of pathogenic microorganisms in chicken broth.

### 3.2. Microbial Inactivation by Heat Treatments in Chicken Broth

To evaluate the contribution of heat to the lethal effect of the combined UV-H treatment, experiments on the heat resistance of all the investigated microorganisms were carried out also in chicken broth. The resulting survival curves did not follow first order kinetics and presented an initial lag phase (shoulder).* E. coli* was the only exception, showing an inactivation curve with a log-linear behavior. The inactivation data were fitted by Geeraerd's model (primary model; (1)). The resulting values for the heat resistance parameters (Sl and *K*_max⁡_) were included in Table 3. As observed, Sl values decreased with temperature, becoming zero at the highest temperatures. That is, the shoulders disappeared proportionate to temperature, as was observed in the UV-H treatments. The relationship between Sl and temperature also followed the Weibullian distribution and was described by Albert and Mafart's equation (2). In the case of *K*_max⁡_ values, they increased with temperature following an exponential relationship. These relationships (secondary models) allowed for the obtention of the corresponding kinetic parameters showed in Table 4. As observed, the shoulder length (Sl value) was higher for* S. aureus*. However, it rapidly decreased with temperature differently to* L. monocytogenes*, which was less influenced by the temperature (*P* value). Concerning the relationships of the inactivation rate, *K*_max⁡_ values of* E. coli* and* Salmonella* Typhimurium showed a similar thermodependence (no significant differences among the slope values), although it was somewhat higher than those of both Gram positive microorganisms, whose thermodependence varied in a parallel manner. This means that the velocity of death of* L. monocytogenes and S. aureus* was affected to a lesser extent by temperature changes than that of* E. coli* and* Salmonella* Typhimurium.

To predict the heat inactivation in chicken broth for each studied microorganism, final equations were developed by including the obtained secondary models for Sl and *K*_max⁡_ in Geeraerd's equation (primary model). When comparing the observed inactivation data at different times and temperatures from those predicted by the final equations, *R* ^2^ values ranged from 0.932 to 0.987, RMSE from 0.158 to 0.386, *A*_*f*_ from 1.132 to 1.444, and *B*_*f*_ from 0.830 to 1.207, indicating an adequate goodness of the fits. Similar to the UV-H final equations, the developed final equations for heat would be adequate to compare the heat resistance of the different microorganisms; define the process criteria to achieve a certain performance criteria; and compare the microbial lethal effectiveness of both the heat and UV-H treatment processes.

### 3.3. Process Criteria for 5-Log_10_ Reductions of Foodborne Microbial Pathogens in Chicken Broth

**Figure 4:**
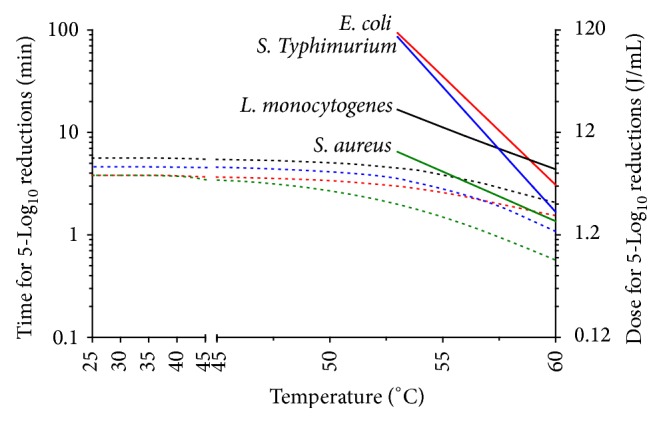
Times and UV doses required to achieve 5-Log_10_ reductions by UV-H (dotted lines) and heat treatments (continuous lines) at different temperatures for* E. coli* (red line),* Salmonella* Typhimurium (blue line),* L. monocytogenes* (black line), and* S. aureus* (green line) in chicken broth.

Figure 4 shows the logarithm of the treatment time and UV dose necessary to inactivate 5-Log_10_ cycles of* E. coli*,* Salmonella* Typhimurium,* L. monocytogenes*, and* S. aureus* at temperatures between 25°C and 60.0°C in chicken broth. UV doses displayed on the secondary *OY* axis were calculated from the trend line equation that resulted from plotting the treatment time against the corresponding UV dose for each reactor. In our facility, this relationship was UV dose = 1.0837 ∗ (time) + 0.0213 (*R* ^2^ = 0.999). For comparison reasons, thermal death time (TDT) curves for each microorganism have also been included showing the relationship between the time for 5-Log_10_ reductions and the heating temperature. In the case of the heat treatments, all TDT curves showed a log-linear profile from which *z* values were deduced (temperature increase for reducing the treatment time 10-fold) at 4.7°C, 4.2°C, 10.8°C, and 11.8°C for* E. coli*,* Salmonella* Typhimurium,* L. monocytogenes*, and* S. aureus*, respectively. These values are in the range of what other authors have obtained for fruit juices [30, 36–38], although slightly higher in the case of the Gram positive bacteria. Based on these data,* E. coli* was the most heat resistant bacterium when the temperature was increased to about 58.8°C, the temperature at which the TDT curves of* E. coli* and* L. monocytogenes* intersected. Above this temperature,* L. monocytogenes* became the most thermotolerant microorganism.

Concerning the UV-H treatments, at temperatures over 50°C the treatment time for 5-Log_10_ reductions of all microorganisms decreased with temperature and followed a concave downward profile. The 5-Log_10_ reductions time varied with the treatment temperature in a way that paralleled that of the thermodependence of *K*_max⁡_ of the UV-H survival curves (Figure 2 and Table 1).* L. monocytogenes* was the most UV-H resistant microorganism at all studied temperatures. UV doses of 6.1 J/mL at room temperature would permit the 5-Log_10_ reduction of any of the investigated pathogenic microorganisms in 5.6 minutes, a level of inactivation that is difficult to achieve with other technologies at the investigated temperatures, treatment times, and energy costs in products like broths. The UV dose and time required to achieve 5-Log_10_ reductions in the target pathogen (*L. monocytogenes*) by UV treatment at room temperature would be reduced by 19.7% at 53°C. A more radical improvement was attained by raising the temperature to 55.0, 57.5, and 60.0°C at which the UV dose and time needed to achieve the performance criterion for* L. monocytogenes* was reduced by 30.1, 44.8, and 62.9%, respectively.

When comparing UV-H and heat treatments, the application of UV-C light at moderate temperatures noticeably reduced the heat treatment time for 5-Log_10_ reductions of tested microorganisms in chicken broth. This reduction in time was greater the lower the temperature of the UV-H treatment was applied. When UV-H treatments were applied at 53°C, 4.5 and 93.5 minutes of the UV-C and heat treatments would be required, respectively, that is, a 20-fold time reduction of the heat processing time. This time reduction of the heat treatments was of 9-, 4-, and 2-fold when the temperature was 55.0, 57.5, and 60.0°C, respectively. Over 60°C, the microbial inactivation due to heat would be greater, thereby reducing the synergistic lethal contribution of UV light and achieving a temperature over which inactivation would be only due to heat.

### 3.4. Conclusions

This study has investigated the lethal microbial effects of UV-C, heat, and UV-H on the different pathogenic microorganisms of reference (*E. coli*,* Salmonella* Typhimurium,* L. monocytogenes*, and* S. aureus*) when treated in chicken broth. Mathematical equations were developed that enabled a comparison between the investigated microorganisms' levels of resistance to the different applied technologies. This defined the target microorganism and established the process criteria (UV dose, time, and temperature) required for 5-Log_10_ reductions of the four pathogens in chicken broth. With all technologies, 5-Log_10_ reductions were obtained for all investigated microorganisms. However, depending on the technology and the target microorganisms, the required time to achieve that performance criterion noticeably varied. For UV treatments (at room or at moderate temperatures),* L. monocytogenes* was the most resistant microorganism, but for heat treatments up to temperatures of 59°C,* E. coli* was. Over this temperature,* L. monocytogenes* was again the most resistant bacteria in chicken broth. On the other hand, UV-C light applied even at room temperature resulted in a technology to control foodborne pathogens in chicken broth that was very promising. However, the time of treatment at this temperature could possibly be too long from a practical point of view. The application of UV-H permitted a reduction in this processing time, which happened to be 20- to 2-fold lower than the corresponding to heat treatments at the same temperature and with an extra energetic cost varying from 6.10 J/mL to 2.26 J/mL. These results indicated that combining UV-C light with mild temperatures permitted a certain performance criterion to be achieved by means of lower UV doses and treatment times than those needed for UV or heat treatments applied to chicken broth alone. The fact that this food product is not considered suitable for being treated by UV light due to its high turbidity and absorption coefficient enhances the importance of the aforementioned results.

## Figures and Tables

**Table 1:** Resistance parameters (Sl and *K*_max⁡_) obtained from the fit of UV-H survival curves of *E. coli*, *Salmonella* Typhimurium, *S. aureus*, and *L. monocytogenes *at different temperatures in chicken broth to Geeraerd's model (1). Estimated standard deviations (SD) of the means are in parentheses. Letters a, b, c, and d indicate statistically significant differences (*P* ≤ 0.05) among Sl and *K*_max⁡_ values of UV-H survival curves of different microorganisms at the same treatment temperature.

Microorganism	Temperature (°C)	Sl (min)	*K*_max⁡_ (min^−1^)	RMSE	*R* ^2^
*Escherichia coli *	25.0	0.67 (0.09)^a^	3.66 (0.34)^a^	0.152	0.994
	50.0	0.68 (0.15)^a^	4.31 (0.22)^a^	0.185	0.993
	52.5	0.55 (0.16)^a^	4.59 (0.20)^a^	0.228	0.990
	55.0	0.33 (0.11)^a^	5.06 (0.34)^a^	0.229	0.991
	57.5	0.10 (0.06)^a^	6.00 (0.43)^a^	0.329	0.985
	60.0	0.00 (0.00)^a^	7.33 (0.62)^a^	0.153	0.996

*Salmonella *Typhimurium	25.0	0.68 (0.04)^a^	2.52 (0.01)^b^	0.098	0.995
	50.0	0.65 (0.10)^a^	3.58 (0.27)^b^	0.144	0.994
	52.5	0.45 (0.02)^a^	3.78 (0.04)^b^	0.140	0.996
	55.0	0.30 (0.10)^a^	4.91 (0.05)^a^	0.219	0.992
	57.5	0.09 (0.05)^a^	5.91 (0.27)^a^	0.261	0.991
	60.0	0.00 (0.00)^a^	10.47 (0.74)^b^	0.256	0.993

*Listeria monocytogenes *	25.0	1.08 (0.13)^b^	2.55 (0.02)^c^	0.077	0.996
	50.0	1.00 (0.02)^a^	2.70 (0.02)^c^	0.068	0.997
	52.5	0.89 (0.04)^b^	3.24 (0.32)^c^	0.212	0.988
	55.0	0.73 (0.13)^b^	3.68 (0.32)^b^	0.212	0.988
	57.5	0.20 (0.05)^b^	4.20 (0.03)^b^	0.308	0.977
	60.0	0.00 (0.00)^a^	5.60 (0.42)^c^	0.253	0.989

*Staphylococcus aureus *	25.0	0.59 (0.17)^a^	3.37 (0.35)^d^	0.204	0.987
	50.0	0.41 (0.09)^a^	5.41 (0.15)^d^	0.222	0.995
	52.5	0.38 (0.01)^a^	6.50 (0.10)^d^	0.279	0.991
	55.0	0.25 (0.02)^c^	7.71 (0.55)^c^	0.213	0.995
	57.5	0.10 (0.00)^a^	11.93 (0.24)^c^	0.3521	0.988

**Table:** (a) Shoulder length secondary model

Microorganism	*δ*	*p*	Sl_0_	Sl_res_	*R* ^2^	RMSE
*Escherichia coli *	56.81 (0.28)^a^	21.46 (2.99)^a^	0.70 (0.02)^a^	0.00 (0.02)^a^	0.996	0.027
*Salmonella *Typhimurium	56.69 (0.42)^a^	16.52 (2.76)^b^	0.70 (0.03)^a^	0.00 (0.02)^a^	0.992	0.034
*Listeria monocytogenes *	57.05 (0.16)^a^	24.06 (2.71)^a^	1.05 (0.03)^b^	0.00 (0.02)^a^	0.998	0.032
*Staphylococcus aureus *	57.45 (0.70)^a^	12.18 (1.72)^b^	0.57 (0.02)^c^	0.00 (0.03)^a^	0.993	0.025

**Table:** (b) *K*_max⁡_ secondary model

Microorganism	*δ*	*p*	*K*_max0_	*R* ^2^	RMSE
*Escherichia coli *	69.22 (0.39)^a^	8.39 (0.57)^a^	3.70 (0.07)^a^	0.998	0.071
*Salmonella *Typhimurium	63.11 (1.27)^b^	10.95 (3.44)^a^	2.76 (0.39)^bc^	0.981	0.501
*Listeria monocytogenes *	67.08 (0.79)^a^	9.44 (1.68)^a^	2.52 (0.14)^b^	0.989	0.151
*Staphylococcus aureus *	62.25 (1.68)^b^	8.02 (2.25)^a^	3.49 (0.53)^ac^	0.985	0.546

**Table 3:** Resistance parameters obtained from the fit of heat inactivation data of *E. coli*, *Salmonella *Typhimurium, *S. aureus*, and *L. monocytogenes *at different temperatures in chicken broth by Geeraerd's model (1). Estimated standard deviations (SD) of the means are shown in parentheses. Letters a, b, c, and d indicate statistically significant differences (*P* ≤ 0.05) among Sl and *K*_max⁡_ values of heat survival curves for each microorganism at different temperatures.

Microorganism	Temperature (°C)	Sl (min)	*K*_max⁡_ (min^−1^)	*R* ^2^	RMSE
*Escherichia coli *	55.6	—	0.52 (0.02)^a^	0.981	0.204
	58.1	—	1.33 (0.04)^b^	0.993	0.203
	60.6	—	3.65 (0.11)^c^	0.991	0.122
	62.6	—	17.49 (1.02)^d^	0.992	0.156

*Salmonella *Typhimurium	55.6	0.41 (0.71)^a^	0.49 (0.11)^a^	0.994	0.024
	58.1	0.19 (0.12)^b^	3.00 (0.16)^b^	0.993	0.196
	60.6	0.00 (0.00)^c^	7.03 (0.66)^c^	0.995	0.188
	62.6	0.00 (0.00)^c^	27.97 (1.23)^d^	0.994	0.157

*Listeria monocytogenes *	55.6	0.80 (0.15)^a^	1.27 (0.11)^a^	0.995	0.105
	58.1	0.81 (0.27)^a^	2.22 (0.23)^b^	0.983	0.153
	60.6	0.80 (0.19)^a^	3.47 (0.11)^c^	0.992	0.204
	62.6	0.47 (0.00)^b^	5.81 (0.15)^d^	0.995	0.165

*Staphylococcus aureus *	53.1	1.30 (0.04)^a^	2.24 (0.03)^a^	0.996	0.082
	55.6	0.12 (0.09)^b^	3.48 (0.04)^b^	0.983	0.204
	58.1	0.00 (0.00)^c^	5.93 (0.31)^c^	0.995	0.163

**Table:** (a) Shoulder length secondary model

Microorganism	*δ* (min)	*p*	Sl_0_ (min)	Sl_res_ (min)	*R* ^2^	RMSE
*Escherichia coli *	—	—	—	—	—	—
*Salmonella *Typhimurium	59.00 (0.07)^a^	63.00 (7.23)^a^	0.43 (0.00)^a^	0.00 (0.02)^a^	0.999	0.048
*Listeria monocytogenes *	63.80 (0.11)^b^	39.04 (3.46)^b^	0.87 (0.01)^b^	0.00 (0.02)^a^	0.998	0.099
*Staphylococcus aureus *	55.26 (0.03)^c^	96.67 (8.66)^c^	1.32 (0.00)^c^	0.00 (0.03)^a^	0.999	0.063

**Table:** (b) *K*_max⁡_ secondary model

Microorganism	Slope (min^−1^)	Intercept (min)	*R* ^2^	RMSE
*Escherichia coli *	0.212 (0.027)^a^	−12.135 (1.602)^a^	0.969	0.425
*Salmonella *Typhimurium	0.240 (0.024)^a^	−13.602 (1.405)^a^	0.981	0.088
*Listeria monocytogenes *	0.093 (0.004)^b^	−5.042 (0.244)^b^	0.996	0.018
*Staphylococcus aureus *	0.084 (0.021)^b^	−4.138 (0.121)^b^	0.999	0.009
